# *MeWRKY IIas*, Subfamily Genes of *WRKY* Transcription Factors From Cassava, Play an Important Role in Disease Resistance

**DOI:** 10.3389/fpls.2022.890555

**Published:** 2022-06-02

**Authors:** Shousong Zhu, Ruochen Fan, Xi Xiong, Jianjun Li, Li Xiang, Yuhui Hong, Yiwei Ye, Xiaofei Zhang, Xiaohui Yu, Yinhua Chen

**Affiliations:** ^1^Hainan Key Laboratory for Sustainable Utilization of Tropical Bioresource, College of Tropical Crops, Hainan University, Haikou, China; ^2^School of Bioengineering, Dalian University of Technology, Dalian, China; ^3^CGIAR Research Program on Roots Tubers and Bananas (RTB), International Center for Tropical Agriculture (CIAT), Cali, Colombia

**Keywords:** WRKY, cassava, *Xam*, pathogen defense, cassava bacterial blight

## Abstract

Cassava (*Manihot esculenta* Crantz) is an important tropical crop for food, fodder, and energy. Cassava bacterial blight (CBB) caused by *Xanthomonas axonopodis* pv. *manihotis* (*Xam*) occurs in all cassava growing regions and threatens global cassava production. WRKY transcription factor family plays the essential roles during plant growth, development, and abiotic or biotic stress. Particularly, previous studies have revealed the important role of the group IIa *WRKY* genes in plant disease resistance. However, a comprehensive analysis of group IIa subfamily in cassava is still missing. Here, we identified 102 *WRKY* members, which were classified into three groups, I, II, and III. Transient expression showed that six *MeWRKY IIas* were localized in the nucleus. *MeWRKY IIas* transcripts accumulated significantly in response to SA, JA, and *Xam*. Overexpression of *MeWRKY27* and *MeWRKY33* in *Arabidopsis* enhanced its resistance to *Pst* DC3000. In contrast, silencing of *MeWRKY27* and *MeWRKY33* in cassava enhanced its susceptibility to *Xam*. Co-expression network analysis showed that different downstream genes are regulated by different *MeWRKY IIa* members. The functional analysis of downstream genes will provide clues for clarifying molecular mechanism of cassava disease resistance. Collectively, our results suggest that *MeWRKY IIas* are regulated by SA, JA signaling, and coordinate response to *Xam* infection.

## Introduction

WRKY is a type of plant-specific transcription factors, which was first reported in sweet potato (*Ipomoea batatas*) as SWEET POTATO FACTOR1 (SPF1) (Ishiguro and Nakamura, 1994), and subsequently, the homologs were isolated from wild oat (*Avena fatua*) (Rushton et al., 1995) and parsley (*Petroselinum crispum*) (Rushton et al., 1996). WRKY transcription factors contain the conserved WRKY domain, which was defined by approximately 60 conserved amino acid sequence WRKYGQK at its N-terminal end, together with a novel zinc-finger-like motif (Eulgem et al., 2000). They have been reported to be involved in essential regulatory functions in multitude of processes during plant growth and development (Riechmann and Ratcliffe, 2000; Yu et al., 2012; Han et al., 2014). However, the most remarkable function of WRKY regulators is their response to diverse abiotic stresses (drought, salt, temperature, waterlogging, and ultraviolet stresses) and biotic stresses (Chen et al., 2018), especially the microbial invaders. Because of the important regulatory function, increasing numbers of *WRKY* genes have been isolated in various species including 74 members in *Arabidopsis* (Kalde et al., 2003; Wang et al., 2011), 116 members in *Gossypium hirsutum* (Dou et al., 2014; Gu et al., 2018), 103 members in *Oryza sativa* (Ramamoorthy et al., 2008), 58 members in *Ricinus communis* (Zou et al., 2016), 81 members in *Solanum lycopersicum* (Huang et al., 2012), 55 members in *Vitis vinifera* (Guo et al., 2014), 97 members in *Actinidia* (Jing and Liu, 2018), 100 members in *Populus* (Jiang et al., 2014), 55 members in *Cucumis sativus* (Ling et al., 2011), 119 members in *Zea mays* (Wei et al., 2012), and 85 members in *Manihot esculenta* (Wei et al., 2016).

WRKY proteins play dual roles in plant pathogen defense, mostly as positive and negative regulators. For example, WRKY functions as a resistance protein to *Ralstonia solanacearum* (Deslandes et al., 2002), which indicated its positive effect in bacteria defending process. It has been reported that bacterial effectors PopP2 and AvrRps4 interacted with WRKY domain-containing proteins, suggesting that these effectors interfered with the WRKY-dependent defense (Sarris et al., 2015). Also, the expression of pepper *CaWRKY40* was induced by *R. solanacearum*, and it promoted the resistance against *R. solanacearum* in pepper (Dang et al., 2013). Another pepper WRKY gene, *CaWRKY1*, was strongly induced by several pathogen infections as well; however, it acted as a negative regulator in pathogen defense processes and inhibited the expression of pathogenesis-related genes (Oh et al., 2008). *WRKY* genes that functioned as negative regulators often prevented the exaggerated defense response (Birkenbihl et al., 2017). Therefore, multiple *WRKY* genes worked together to form a transcriptional network with positive and negative feedback loops and feed-forward modules to keep the defense response in a moderate range (Eulgem and Somssich, 2007; Pandey and Somssich, 2009). The conserved structural features of WRKY might be essential to integrate the members in the defense network (Eulgem and Somssich, 2007). The promoters of *Arabidopsis WRKY* genes responding to pathogen and/or SA had a substantial enrichment of W-box (C/T)TGAC(T/C), suggesting that these *WRKY* genes could be auto-regulated or controlled by other WRKY proteins (Dong et al., 2003). Chromatin immune precipitation assays confirmed that WRKY33 bound its own promoter *in vivo*, indicating a potential positive feedback regulatory loop (Mao et al., 2011).

Moreover, WRKY members perform their regulatory functions in diverse ways. Most of the WRKY proteins could bind to the W-box in the promoter of target genes to regulate their expression (Li et al., 2011; Jiang et al., 2012), for example, AtWRKY57 (Jiang et al., 2012) and OsWRKY47 (Raineri et al., 2015). Genome-wide binding site screening revealed that upon the inducement with flg22 (the conserve domain of bacterial flagellin), each of *Arabidopsis* WRKY18, WRKY40 and WRKY33 binds to more than 1,000 gene loci. W-box elements were the most frequently occurring motifs for all three group *WRKY* factors (Birkenbihl et al., 2017). WRKY proteins also bound to WT-box (GGACTTTC) (Kanofsky et al., 2017), PRE4 element (Cai et al., 2008), and WK box (TTTCCAC) (Verk et al., 2008). Another common way for WRKY to play regulatory role was to interact directly with other transcription factors. In grapes, VvWRKY03 acted through a combinatorial effect with VvMYB14, suggesting that these two regulators might interact at the protein level as previously reported in other species (Vannozzi et al., 2018). The target genes regulated by WRKY were involved in various plant life activities, including pathogen-related genes, ET, SA, JA, and ABA-induced pathway genes, and so on (Du and Chen, 2000; Tao et al., 2011; Jiang and Yu, 2016; Birkenbihl et al., 2017; Ullah et al., 2018).

*WRKY IIa* group genes appear to include a small number of members, for example, 3 in *Arabidopsis thaliana* (Eulgem et al., 2000), 4 in *Oryza sativa* (Wu et al., 2005), 6 in *Gossypium hirsutum* (Dou et al., 2014), 5 in *Populus trichocarpa* (He et al., 2012), and 3 in *Cucumis sativus* (Ling et al., 2011), but they participate widely in the regulation of diverse defense processes. *Arabidopsis AtWRKY18, AtWRKY40*, and *AtWRKY60* exhibit a complex pattern of physical and functional interactions in response to the microbial pathogens such as *Pseudomonas syringae* and *Botrytis cinerea* (Xu et al., 2006). *Arabidopsis wrky18 wrky40* and *wrky18 wrky60* double mutants and *wrky18 wrky40 wrky60* triple mutants are more resistant to *P. syringae* but more susceptible to *B. cinerea*. All *OsWRKY IIa* members are also involved in modulating plant innate immunity (Peng et al., 2010). *Hordeum vulgare* HvWRKY1 and HvWRKY2, the homologous proteins of *Arabidopsis* AtWRKY18 and AtWRKY40, interacted with mildew resistance locus A (MLA) to regulate the mediated resistance to *Blumeria graminis f sp. hordei* (Shen et al., 2007).

Cassava (*Manihot esculenta* Crantz) is an important staple crop as a source of food and income for hundreds of millions of people in tropical areas (Chaves et al., 2021). Cassava root starch is widely used in the pharmaceutical, textile, paper, and biofuel industries. This major crop is threatened by several pathogens, especially the vascular and systemic *Xanthomonas axonopodis* pv. *manihotis* (*Xam*) as its devastating effects on the cassava productivity. In cassava, 85 putative WRKY members have been detected using genome sequence analysis (Wei et al., 2016). An increasing number of evidence has confirmed the important roles of WRKY transcription factors in cassava defense processes, and several *MeWRKY* genes have been identified to be involved in fighting against microbial invaders. For example, MeWRKY20 regulates disease resistance through physically interacting with MeATG8 (autophagy-related protein 8) a/f/h and transcriptional activation *MeATG8a* (Yan et al., 2017). Similarly, MeWRKY75 was also capable of positively regulating resistance against bacterial blight by forming protein complex with MeWHY1/2/3 and activating the expression of MeWHY3 (Liu et al., 2018). However, the roles of majority MeWRKY family members remain poorly understood.

In this study, we comprehensively identified 102 typical WRKY members containing the WRKY domain when performing whole-genome scan using conserved WRKY domain sequence as a query. We then focused on six WRKY members from Group IIa and further determined their roles in cassava disease resistance. The six *MeWRKY IIas* were self-activated and could bind to W-box. The expression of *MeWRKY IIas* responded to the treatments of SA, JA, and *Xam*. Moreover, *MeWRKY IIa* members *MeWRKY27* and *MeWRKY33* positively regulate disease resistance against CBB. Thus, WRKY group IIa members *MeWRKY27* and *MeWRKY33* are involved in bacteria defense in cassava and can be considered as the target genes for resistance to CBB.

## Materials and Methods

### Plant Materials and Growth Conditions

The cassava cultivar SC8 (South China 8) and *Arabidopsis* Col-0 conserved by our laboratory were used in this study. Cassava plants were planted in green house condition (28°C, 12-h day/12-h night cycle, 120–150 μmol m^−2^ s^−1^ light intensity, and 80% humidity). *Arabidopsis* was planted under 16-h light/8-h dark at 22°C.

### Identification and Comprehensive Analyses of MeWRKYs

The conserved hidden Markov model (HMM) profile of WRKYGQK domain (PF03106) of WRKY proteins downloaded from Pfam (http://pfam.xfam.org/) was used as a query for BLAST against all protein sequences of cassava genome v6.0 in Phytozome database (https://phytozome.jgi.doe.gov/pz/portal.html) using HMMER3.0 software (http://hmmer.janelia.org/) with e-value threshold of 1e-10. Then, the cassava-specific HMM file for the WRKY family was constructed by hmmbuild from the aligned results of the initially obtained WRKY protein sequences and used for second round HMM searches against cassava genome. The candidate protein sequences were further identified for the presence of WRKY domain by Pfam and SMART database (http://smart.embl.de/). The MeWRKY proteins were named based on their position on cassava chromosome. In addition, the physical and chemical properties of MeWRKY proteins were analyzed using online software ProtParam (http://web.expasy.org/protparam/). The subcellular locations of MeWRKY proteins were predicted with WoLF PSORT (https://wolfpsort.hgc.jp/). Moreover, the multi-sequence alignment of MeWRKYs was aligned using the ClustalW (Thompson et al., 2003), and the phylogenetic tree was constructed using MEGA7 (Kumar et al., 2016). Genome annotation files of cassava were downloaded from Phytozome database, and gene structure was analyzed using GSDS (http://gsds.gao-lab.org). The conserved protein motif analysis was carried out using online software MEME (http://gsds.gao-lab.org). All identified motifs were annotated using InterProScan (http://www.ebi.ac.uk/Tools/pfa/iprscan/). The gene structure, conserved motifs, and the phylogenetic tree of MeWRKYs were combined using online tool iTOL (Letunic and Bork, 2006). Subfamilies were further identified and named according to the genetic relationship among different clades and the conserved protein domain composition.

Chromosomal locations of *MeWRKYs* were retrieved from cassava genome annotation file and visualized using TBtools (Chen et al., 2020a). Gene clusters are defined as a single chromosome containing two or more genes within 200 kb (Holub, 2001). All coding proteins in cassava were first aligned using BLASTP (E-value cutoff = 1e-10) against itself. Additionally, the BLASTP hit results were then compiled as the input for MCScanX (Wang et al., 2012) to perform gene duplication and collinearity analysis with default parameters.

Public cassava RNA-seq data for cassava tissues or cassava infected with *Xam* were obtained from the high-throughput DNA and RNA sequence read archive (SRA) of the NCBI. Analysis of *MeWRKY* gene expression profiles was performed as previously described (Hong et al., 2021). Heatmaps of *MeWRKYs* were processed based on log2-transformed FPKM (fragments per kb per million fragments) values and visualized using TBtools.

### Subcellular Localization of *MeWRKY IIas*

To identify the subcellular location of *MeWRKY IIas*, the full-length cDNA sequences of six *MeWRKY IIas* were cloned and linked into pGBT vector and then transferred into cassava protoplast (Wu et al., 2017). The cell nucleus was stained with Hoechst 33,342 solution (Solarbio, Beijing, China). The green fluorescent signals were observed with confocal laser-scanning microscope (TCS SP8, Leica, Heidelberg, Germany).

### Hormones Treatment and *Xam* Inoculation

The *in vitro* cassava plantlets were transferred to nutrition pots. After 2 months, cassava plants were sprayed with 100 of μmol/L MeJA (methyl jasmonate) and 100 of μmol/L SA (salicylic acid) (both dissolved in 1: 9, v; v ethanol). Mock plants were sprayed with 10% ethanol (1: 9, v; v). Cassava leaves were sampled at 0, 15, 30, and 60 min after the hormone treatment. For the pathogen treatment, the single clone of *Xam*CHN11 on LPGA solid medium was transferred into 5 ml of liquid medium and cultivated at 28°C for another 48 h (Li et al., 2018). Then, the bacteria were collected by centrifugation. The bacterial suspensions (OD_600_ = 0.1, 1 × 10^8^ CFU ml^−1^) were prepared with sterile 10 mmol/L of MgCl_2_ and then infiltrated into lower leaves using a 1-ml needleless syringe. Cassava leaves were sampled at 0, 5 h and 1, 2, 4, 8, and 15 days. At least three repetitions were employed for each treatment.

### RNA Isolation and Quantitative Real-Time PCR (qRT-PCR)

Total RNA isolation and first-strand cDNA synthesis were performed using RNAprep Pure Plant Kit (Polysaccharides & Polyphenolics-rich) (TIANGEN, DP441, Beijing, China) and RevertAid First-Strand cDNA Synthesis Kit (Thermo Scientific, K1622, Waltham, MA, USA) according to the manufacturer's instructions. The qRT-PCR was performed using the TB Green^TM^ Premix Ex Taq^TM^ II (TIi RNaseH Plus) (TaKaRa, RR820A, Dalian, China) to detect the expression level of target genes. *MeUBQ10* (Phytozome: Manes.07G019300) was used as an internal reference gene for qPCR studies. Relative quantification of gene transcription level was analyzed using the comparative threshold cycle 2^−Δ*ΔCT*^ method. The gene-specific primers for qPCR analysis are listed in Supplementary Table 1.

### Yeast One-Hybrid Assay of MeWRKY IIas With W-Box

To identify the transcriptional activation of *MeWRKY IIas*, the full-length cDNA sequences of six *MeWRKY IIa* members were cloned into pGBKT7 vectors and then transferred to Y2HGold yeast strains. After confirmed by PCR, the positive yeast clones were cultivated in SD/-Trp liquid medium at 28°C until OD_600_ reached 0.6. The transformants were diluted into different concentrations and selected on the SD/-Trp, SD/-Trp/X-α-gal, SD/-Trp/X-α-gal/AbA (aureobasidin A), and SD/-Trp-His-Ade deficiency medium. The transcriptional activities were assessed according to the yeast growth status after 2–3 days in an incubator at 28°C.

To assess the combining capacity of MeWRKY IIa members with W-box, yeast one-hybrid assay was performed. W-box was cloned into pBait-AbAi vector and transferred into Y1HGold yeast strain according to the manual of Yeast maker Yeast Transformation System 2 (PT1172-1, Clontech Laboratories, Inc. A Takara Bio Company, CA 94043). After culturing on the SD/-Ura solid medium for about 2–4 days, the positive clones were identified and cultivated in liquid medium until OD_600_ = 0.6. Then, the diluted yeast culture was dotted on SD/-Ura solid medium containing 0, 100, 200, 300, 400, 500, 600, 700, and 800 ng/ml AbA. After 3–5 days, the growth status of yeast colony was observed and the minimum inhibited concentration of AbA was determined.

The full-length cDNA sequence of *MeWRKY IIas* was cloned into pGADT7 vector, individually. Then, the plasmid of pGADT7-*MeWRKY IIas* was transferred into the Y1HGold yeast strain containing pAbAi-W-box. After cultivating on SD/-Leu solid medium at 28°C for about 2–4 days, the positive clones were identified by PCR and then cultivated in SD/-Leu liquid medium until OD_600_ = 0.6. The yeast culture was diluted by 10, 100, and 1,000 times as well as the control (pABAi-*p53*+pGADT7-*Rec*). The dilution was dotted on SD/-Leu solid medium containing different AbA concentrations. The interaction between MeWRKY IIas and W-box was assessed by the growth performance of transformant.

### Generation of *MeWRKY IIas* Overexpressed *Arabidopsis* Plants and Pathogen Sensitivity Test

A number of six *MeWRKY IIas* full-length cDNA sequences were cloned into plant expression vector pEGAD. The positive plasmids and empty vector were transferred to *Agrobacterium tumefaciens* GV3101 and cultivated in YEB liquid medium at 28°C for about 18 h. Then, the bacteria precipitation was collected and resuspended in 5% sucrose solution containing 0.1% silwet L-77, and the concentration was adjusted to OD_600_ = 1.0. The transgenic *Arabidopsis* plants were generated through *Agrobacterium*-mediated floral dipping method, and the positive transformants were screened by 0.1% Basta and PCR amplification. The homozygotes of T3 plants were used for detecting the *Pst* DC3000 sensitivity. Then, 4-week-old transgenic *Arabidopsis* plants were inoculated with *Pst* DC3000 as previously described (Huang et al., 2021).

### Virus-Induced Gene Silencing in Cassava and *Xam* Sensitivity Test

To analyze the functions of *MeWRKY IIa* genes in cassava, the loss-of-function plants were created *via* VIGS method (Tuo et al., 2021). The regions of target genes for genome-wide off-target gene silencing were selected using SGN VIGS Tool (Fernandez-Pozo et al., 2015). The specific primer pairs of *MeWRKY IIas* fragment sequences for VIGS are listed in the Supplementary Table 1. The amplified fragments were cloned into pCsCMV-NC using the Nimble cloning methods (Yan et al., 2019). pCsCMV-*ChlI*_345_ (345 bp *magnesium chelatase subunit I* fragment) was used as the positive control and pCsCMV-NC as the negative control. All the vectors were transformed into the *Agrobacterium tumefaciens* GV3101 with pSoup-p19 helper plasmid. The leaves of cassava plantlets at 8 weeks after planting were injected with 100 μl *Agrobacterium* containing recombinant plasmid (OD_600_ = 0.8). Injections were performed at 8–10 spots (10 μl agrobacterium suspension for each spot) on both sides of the main vein per leaf to enlarge the infiltrated leaf area. When the positive plants exhibited apparent photobleaching in the veins of leaves, the silencing effect of target gene was detected by real-time fluorescence quantitative PCR. Leaves of silenced plants were inoculated with *Xam*CHN11 pathogen (OD_600_ = 0.1 or 0.01). Samples were taken at 6 days after inoculation for lesion area investigation. The lesion areas were measured using ImageJ 1.51 (Schneider et al., 2012). The bacterial growth in cassava plants was measured as previously described (Medina et al., 2018). All experiments were taken three times showing similar results.

### Co-expression Analysis of *MeWRKY IIas* and Identification of *MeWRKY IIas*-Regulating Genes

To find the *MeWRKY IIas*-regulating genes, the 2-kb upstream sequences (putative promoter regions) of “ATG” of total 33,033 genes in the cassava genome were extracted and analyzed with TSSP. The conserved W-box sequence [(T)(T)TGAC(C/T)] in promoter regions was used as a marker to identify *MeWRKY*-regulated gene candidates possibly involved in plant disease resistance. Co-expression modules were generated for the *MeWRKY IIas* and W-box genes based on the 37 selected transcriptomes (Supplementary Table 2). The network was analyzed with the weighted gene co-expression network analysis (WGCNA) package (Zhang and Horvath, 2005) as previously described (Hong et al., 2021). A pair of *MeWRKY* and W-box genes with a weight value ≥ 0.15 was defined as associated. The gene modules were visualized with Cytoscape (Shannon et al., 2003).

### Statistical Analysis

Data were presented as mean ± standard deviation. Significant difference was analyzed using student's *t*-test. The mean values were considered significantly different when *p* < 0.05. All statistical data were analyzed using SPSS 20.0 software. The measurement values presented were obtained from the means of three biological replicates.

## Results

### Genome-Wide Identification and Evolutionary Analysis of *MeWRKYs*

Genome-wide search using conserved WRKY domain (PF03106) revealed a total of 102 non-redundant candidate *MeWRKY* genes from cassava genome database after manually removing the redundant sequences. We further named these cassava *MeWRKY* members as *MeWRKY1-102* according to their position on chromosomes. The detailed information of each gene is shown in Supplementary Table 3. In general, the total length of predicted cassava MeWRKYs proteins ranged from 115 (MeWRKY37) to 741 (MeWRKY65) amino acid residues. The relative molecular mass (MWs) and the predicted isoelectric points (pIs) of MeWRKYs ranged from 12.63 (MeWRKY4) to 80.43 kDa (MeWRKY65), and from 4.91 (MeWRKY62) to 9.89 (MeWRKY44), respectively. The predicted subcellular localization of MeWRKYs showed that most of them have a great possibility to locate in the nucleus with a few in the chloroplast (MeWRKY3, MeWRKY88), peroxisome (MeWRKY15, MeWRKY20, MeWRKY54), cytoskeleton (MeWRKY21), and cytoplasm (MeWRKY29).

All *MeWRKYs* were located on chromosomes and showed that an uneven distribution pattern except for *MeWRKY100* to *102* were located on the scaffolds. The numbers of the *MeWRKYs* on chromosome (Chr.) ranged from 1 (Chr. 4/Chr. 11) to 18 (Chr. 1), with a mean of 5.5 *MeWRKYs* per chromosome (Supplementary Figure 1). Many *MeWRKYs* tend to distribute at the chromosomal ends. Gene clusters are important for predicting co-expression genes or potential function of clustered genes (Overbeek et al., 1999). A total of thirty *MeWRKYs* were clustered into 11 clusters in cassava genome (Supplementary Figure 1). The gene clusters irregularly distributed on chromosomes. A total of two clusters were located on both Chr. 1 and Chr. 12, and only one cluster was found on each of Chr. 2, Chr. 3, Chr. 5, Chr. 7, Chr. 10, Chr. 14, and Chr. 16.

**Figure 1:**
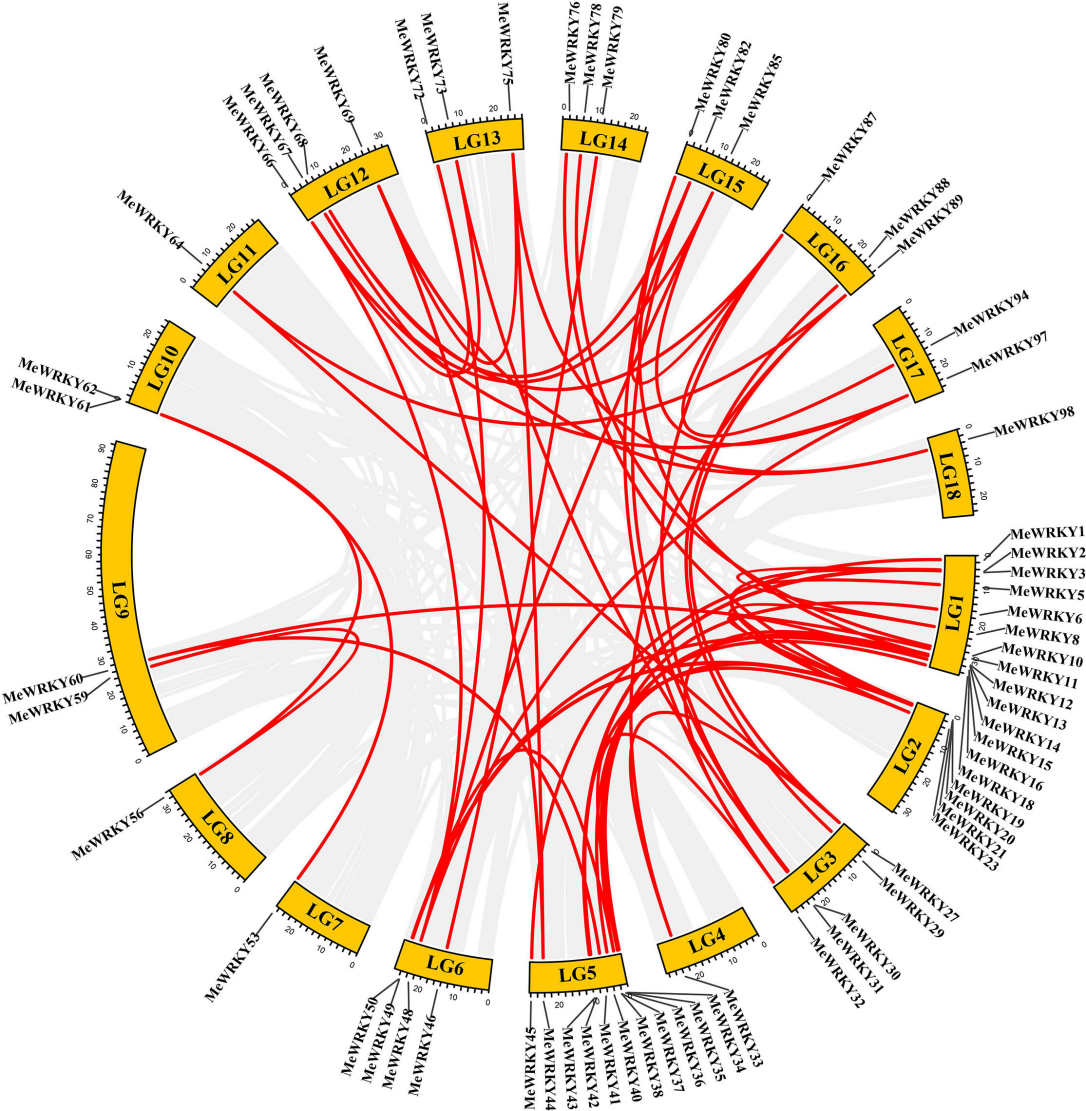
Chromosomal distribution of duplicated *MeWRKYs* pairs generated by MCScanX. Gray lines represent synteny blocks in the cassava genome, and duplicated *MeWRKY* gene pairs are connected with red lines.

Gene duplication events were considered as the main evolutionary force. According to the previous studies, two or more adjacent homologous genes located on a single chromosome were defined as tandem duplicated genes, whereas homologous genes between different genomic regions or chromosomes were regarded as segmental duplication genes (Liu and Ekramoddoullah, 2009). A total of 60 homologous gene pairs involving 65 *MeWRKY* genes, accounting for almost 64% of *MeWRKYs* genes, were identified as segmental duplication genes, whereas only one pair of *MeWRKY* genes (*MeWRKY89* and *90*) was identified as tandem duplication genes (Figure 1; Supplementary Table 4). Among all the segmental duplication pairs, 18 pairs were discovered in subgroup IIc, followed by 8 pairs in subgroups I and IIe, 7 pairs in subgroup IId, 6 pairs in subgroup III, and 5 pairs in subgroup IIa. Subgroup II experienced the majority of segmental duplication events. These results indicated that some *MeWRKYs* might be generated by segmental duplication events, which acted as a major force to drive the evolution of the *MeWRKYs*. The same phenomenon was also found in many other plant *WRKY* families, such as peanut (Song et al., 2016), soybean (Yin et al., 2013), willow (Bi et al., 2016), and carrot (Nan and Gao, 2019).

### Identification of WRKY IIa Group Members Using Conversed Domain Analysis

**Figure 2:**
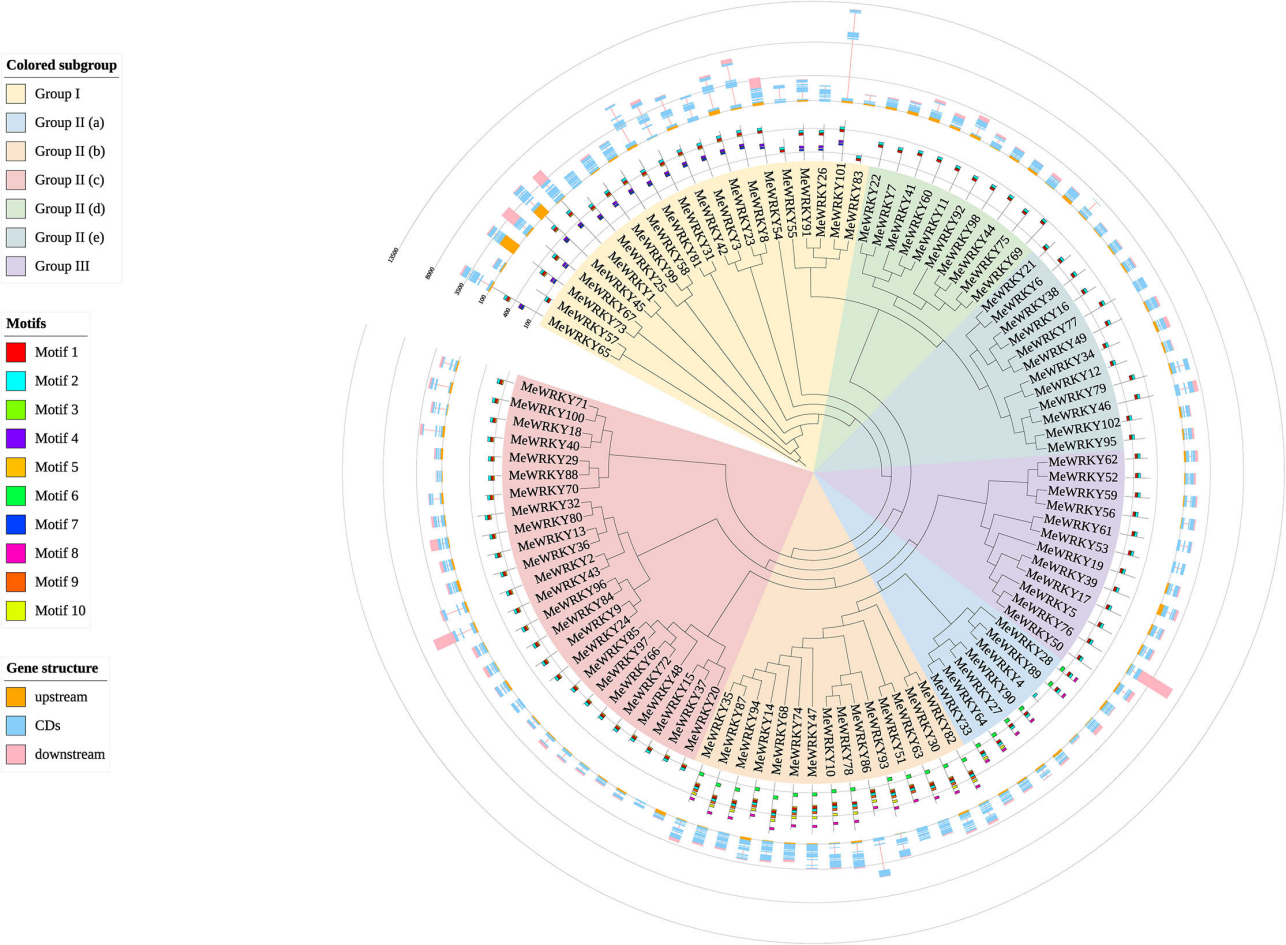
Phylogenetic tree, gene structure, and conserved motifs of 102 MeWRKYs generated from the amino acid sequences with WRKY domains from cassava. The neighbor-joining tree was constructed using MEGA 7.0 with 1,000 bootstraps. The middle circle shows the conserved motifs of the 102 MeWRKYs. The outermost circle shows the exon–intron gene structure of 102 MeWRKYs.

The most prominent structural feature of WRKY proteins is the WRKY domain (WD, a highly conserved hepta-peptide stretch of WRKYGQK at the N-terminus followed by a zinc-finger-like motif) (Eulgem et al., 2000). To better understand the phylogenetic relationship and classification of *MeWRKYs*, neighbor-joining (NJ) phylogenetic tree was generated (Supplementary Figure 2; Figure 2). To identify the variations in WRKY domains, a multiple sequence alignment of the core WRKY domain, spanning about 60 amino acids of all 102 MeWRKYs, is shown in Supplementary Figure 3. The phylogenetic tree and multiple core sequence alignment showed that the 102 MeWRKYs could be divided into three groups based on the number of WRKY domain sequences and the features of the zinc-finger-like motif. The WRKY I and the WRKY III group contained 21 and 12 MeWRKYs members as WRKY I group proteins usually contain two WD sequences and two C2H2 motif (C-X_4−5_-C-X_22−23_-H-X_1_-H), and WRKY III group proteins usually contain C2-H-C motif (C-X_7_-C-X_23_-H-X_1_-C) in addition to one WRKY domain. Generally speaking, group I contained two WRKY domains (an N-terminal and a C-terminal WRKY domain), whereas MeWRKY55, 57, and 83 only contain a C-terminal WRKY domain. Besides, MeWRKY57 contains C2-H-C motif (C-X_7_-C-X_23_-H-X_1_-C), but we still classify it to group I according the phylogenetic tree with *Arabidopsis* WRKY members (Supplementary Figure 2). The WRKY II group proteins usually contain only one WRKY domain and same type of zinc-finger-like motif with WRKY I group. According to sequence variances in zinc-finger-like motif, WRKY II proteins can be divided into five subgroups IIa [CX_5_CPVKKK(L/V)Q], IIb (CX_5_CPVRKQVQ), IIc (CX_4_C), IId (CX_5_CPARKHVE), and IIe [CX_5_CPARK(Q/M)V(E/D)] with 7, 15, 25, 10, and 12 WRKY members, respectively. The highly conserved WRKYGQK domain was present in 97 MeWRKY members, whereas a group I (MeWRKY57) and three group IIc members (MeWRKY29, 70, and 88) have WRKYGQR and WRKYGKK domains, respectively. The group IIa member MeWRKY4 was observed to have lost its partial WRKY domain. The slight variations in WRKYGQK domain were also found in other plant species, such as carrot (Nan and Gao, 2019), pineapple (Xie et al., 2018), cucumber (Chen et al., 2020b), pepper (Zheng et al., 2019), and tomato (Huang et al., 2012). The variations in WRKY domain may relate to the binding specificity to W-box cis-elements (Guo et al., 2014; Chen et al., 2018). An indirect evidence was that tobacco NtWRKY12, which contains a WRKYGKK domain, could bind to WK-box rather than W-box (Verk et al., 2008). Moreover, the soybean GmWRKY6 and GmWRKY21, which have a WRKYGKK domain, do not bind normally to the W-box (Zhou et al., 2008). These results indicate a high complexity between cassava *MeWRKY* genes.

To explore the structural diversity of *MeWRKYs*, the exon–intron structure analyses of 102 *MeWRKYs* were performed and mapped to the family phylogenetic tree (Figure 2). All *MeWRKYs* have at least 2 exons. The number of exons in *MeWRKYs* ranged from 2 to 6. Among them, 10 of *MeWRKYs* only have 2 exons, 51 of *MeWRKYs* had 3 exons, 14 of *MeWRKYs* had 4 exons, 19 of *MeWRKYs* had 5 exons, and the rest of 8 *MeWRKYs* had 6 exons. All *MeWRKYs* had at least one intron inserted. The PR intron was found in the WRKY domains in group I, IIc, IId, and IIe, whereas the VQR intron distributed in the C2H2 motif of the group IIa and IIb (Supplementary Figure 3). In general, the closest *MeWRKY* genes in the same subfamily have similar gene structure, supporting their close evolutionary relationships.

To further study the characteristic regions of the MeWRKYs, the conserved motifs of the 102 candidate MeWRKYs were detected by the MEME and then annotated with InterProScan (Figure 2, Supplementary Table 5). A total of 10 distinct motifs were identified. Motifs 1 and 2, broadly distributed across MeWRKY proteins, were annotated as WRKY DNA-binding domain. Motifs 4 and 7, also identified as WRKY domain (N-terminal), were only found in group I. The motif 6, which was annotated as leucine zippers (LZ), was found to be specific to subgroups IIa (except for MeWRKY4) and IIb. The similar motif composition of the MeWRKY proteins with each subclass indicates that the protein structure and function were relatively conserved within each specific subfamily.

As plant *WRKY IIa* genes play an important role in plant disease resistance (Xu et al., 2006; Liu et al., 2007; Shen et al., 2007), we next paid further attentions on cassava *MeWRKY IIas*. After aligning the seven cassava WRKY IIa protein sequences, i.e., MeWRKY4, MeWRKY27, MeWRKY28, MeWRKY33, MeWRKY64, MeWRKY89, and MeWRKY90, we observed that all of the WRKY IIas from cassava contained one typical WRKY domain, except for MeWRKY4 that only had the zinc finger motif (Supplementary Figures 3, 4). Thus, we selected the six members of cassava WRKY IIa group as candidates for the follow-up functional analysis. The molecular weights of the six proteins varied from 28.45 (MeWRKY27) to 36.52 kDa (MeWRKY64) and the isoelectric points ranged from 8.26 (MeWRKY89) to 9.01 (MeWRKY64).

### Expression Patterns of *MeWRKYs* in Different Tissues

To assess the potential functions of *MeWRKYs* during cassava growth and development, the expression patterns of all 102 *MeWRKYs* in leaves and different stage roots were investigated using a standard transcriptome analysis procedure based on public transcriptomic data (Supplementary Figure 5). Some *MeWRKYs* showed significantly temporal and spatial differences in expression. For example, *MeWRKY56* exhibited the highest transcript levels in Arg7 leaves. In addition, the expression of several *MeWRKYs*, such as *MeWRKY32, MeWRKY40*, occurred preferentially in roots. However, *MeWRKY6, MeWRKY9, MeWRKY10, MeWRKY14, MeWRKY20*, and so on did not show any detectable expression in the leaves and roots. The expression analysis of the different root developmental stages showed that several genes (*MeWRKY17, MeWRKY19, MeWRKY39, MeWRKY40, MeWRKY44, MeWRKY56, MeWRKY92, MeWRKY98, MeWRKY101*, with FPKM > 20) had higher expression in the early root developmental stage of KU50. These results indicated that *MeWRKYs* may play an important role in the regulation of cassava growth and development.

### Subcellular Localization of *MeWRKY IIas*

**Figure 3:**
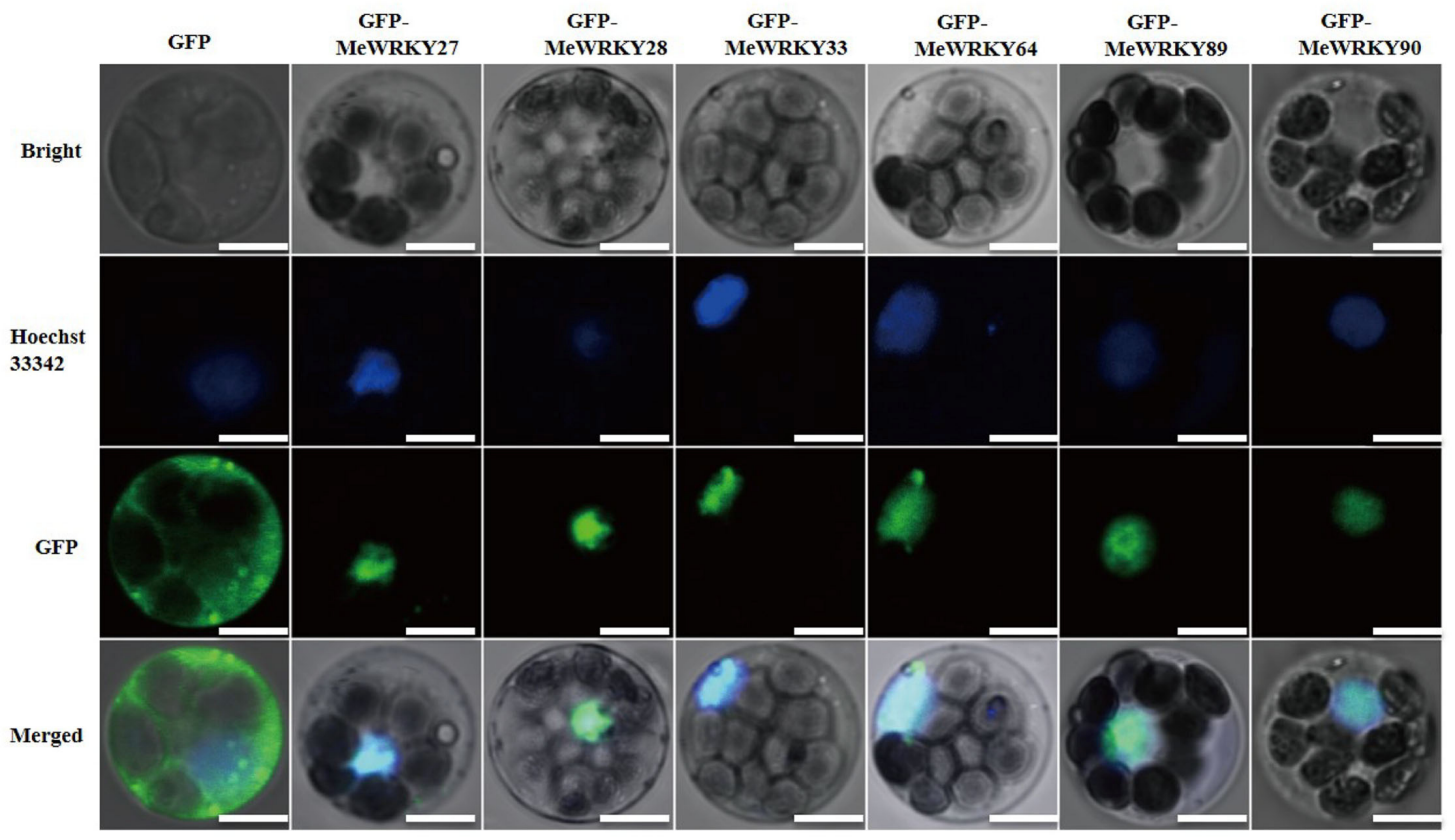
Subcellular localization of MeWRKY IIas in cassava protoplast. The cell nucleus was stained with Hoechst 33342. GFP alone serves as a negative control. Bar = 5 μm.

Subcellular location is important for gene function. All MeWRKY IIas were predicted to locate in nucleus. To further confirm the localization of MeWRKY IIas, the pGBT recombinant vectors carrying *GFP-MeWRKY IIas* were transiently expressed in cassava protoplast. The protoplasts expressing GFP-MeWRKY IIas fusion proteins showed fluorescent signals exclusively restricted to the nucleus, whereas the signal in protoplasts expressing GFP protein was observed in both cell nucleus and cytoplasm (Figure 3). Thus, the MeWRKY IIas were located in cell nucleus.

### Expression Patterns of *MeWRKY IIas* Induced by *Xam* Strains With a Different Virulence

**Figure 4:**
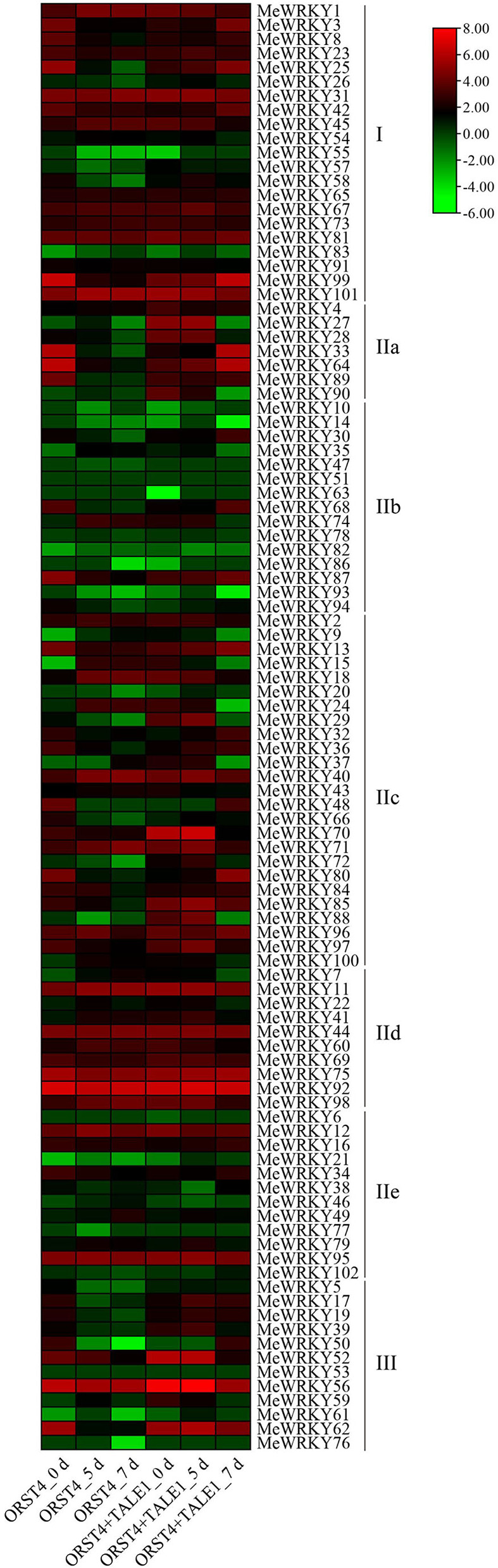
Expression profiles of cassava *WRKYs* in response to different *Xam* (*Xanthomonas axonopodis* pv. *manihotis*) strains. The 12 transcriptome datasets were downloaded from SRA database of NCBI. The transcriptome data were obtained from cassava (MCOL1522) tissue culture seedlings treated with *Xam* strain ORST4 (low pathogenic strain) and ORST4+TALE1 (high pathogenic strain). The color scale represents relative expressions (red: increased transcript abundance; green: decreased transcript abundance).

Transcriptome data of cassava treated with *Xam* strain of ORST4 (low pathogenic strain) and ORST4+TALE1 (high pathogenic strain) were obtained from the National Center for Biotechnology Information (NCBI). Heatmap of expression profile was created to display the relative expression of *MeWRKYs* during *Xam* infection (Figure 4). Most of the cassava *WRKYs* responded to the treatment of *Xam*. For *WRKY IIa* candidates, the expression of *MeWRKY27* was induced and then inhibited by both ORST4 and ORST4+TALE1. Both weak and strong pathogenic strains downregulated the expression of *MeWRKY28* and *MeWRKY90*. The expression of *MeWRKY33* and *MeWRKY64* was reduced by ORST4 infection, but increased when cassava was infected ORST4+TALE1. *MeWRKY89* was repressed by ORST4 and seemed to be not affected by ORST4+TALE1. Thus, the six *WRKY IIa* members were involved in the response to the infection of *Xam*, and they might have different functions in the regulatory network.

### Phytohormone and Pathogen-Induced Differential Expression of *MeWRKY IIas*

**Figure 5:**
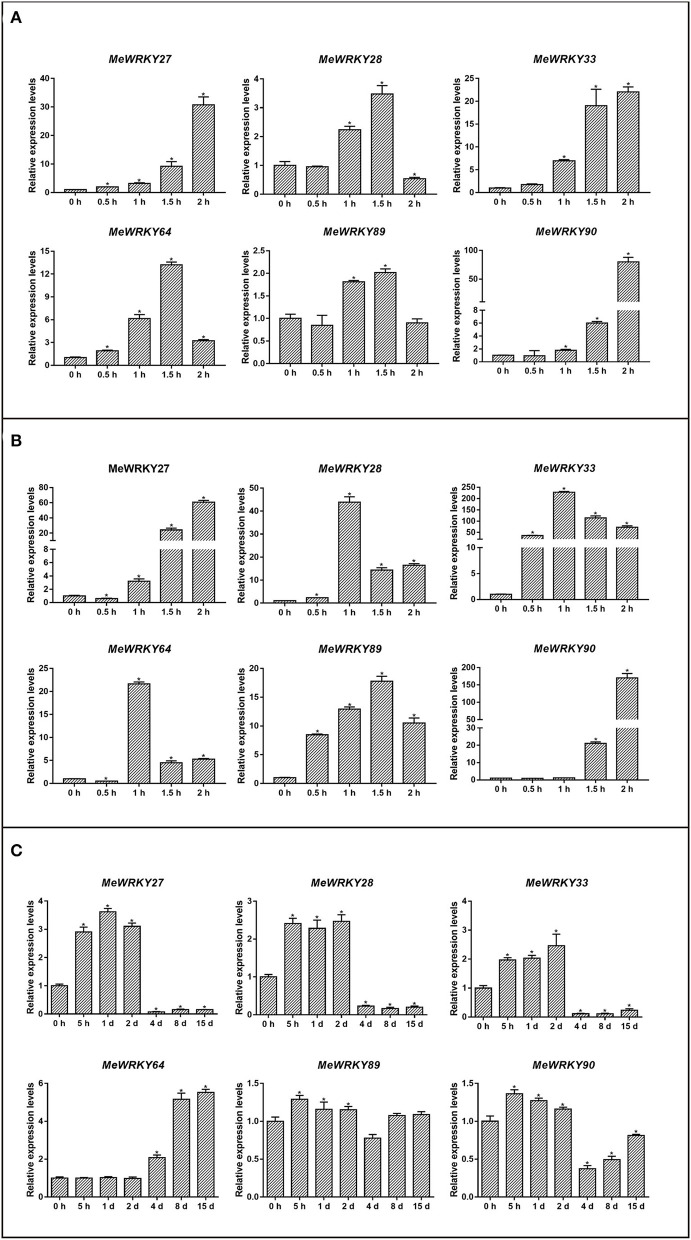
Expression analysis of *MeWRKY IIa* genes in response to JA **(A)**, SA **(B)**, and *Xam* **(C)**. Each value represents the mean ± SD of three replicates. Asterisks indicate a significant difference (*p* < 0.05) between control and treatments.

To determine whether *MeWRKY IIas* are involved in hormone-induced plant immune pathway, we investigated the expression patterns of *MeWRKY IIas* in cassava samples treated with JA and SA. Briefly, six *MeWRKY IIas* were all induced by JA (Figure 5A). Particularly, the expression of *MeWRKY27, MeWRKY33*, and *MeWRKY90* reached >30-, 22-, and 80-fold changes, respectively, at 2 h after the treatment with JA than with control. The expression of *MeWRKY28, MeWRKY64*, and *MeWRKY89* was found to be upregulated at 60 and 90 min, after peaked at 90 min, and then expression of the three genes declined. The six *MeWRKY IIas* were also found to be prominently upregulated with maximum transcript level >15-folds with the treatment of SA (Figure 5B). *MeWRKY27* and *MeWRKY90* displayed similar expression profile that the transcription was rapidly promoted at 90 min and 2 h after treatment and reached the peak value at 2 h. As for *MeWRKY28, MeWRKY33*, and *MeWRKY64*, the presence of SA increased their expression at 60 min and then downregulated the expression. It is a remarkable fact that the transcription peak value of *MeWRKY33* and *MeWRKY90* reached to 230- and 170-folds compared with control.

We also examined the expression profile of *MeWRKY IIas* in cassava leaves infected with *Xam*. The expression of all *MeWRKY IIas* was induced by *Xam* (Figure 5C). Relative expression of *MeWRKY27, MeWRKY28, MeWRKY33, MeWRKY89*, and *MeWRKY90* was significantly promoted at 5 h, 1 d, and 2 d, and after that, the transcription of these genes declined. *MeWRKY64* was an exception with unique expression pattern. The upregulation of *MeWRKY64* started from 4 days and kept high expression level until 15 days. Thus, all the six *MeWRKY IIa* genes were involved in the response to the infection of *Xam*, and the expression of *MeWRKY64* was activated late but lasted for more than 2 weeks at high level.

### MeWRKYIIas Can Combine With W-Box and Activate Report Gene Expression

**Figure 6:**
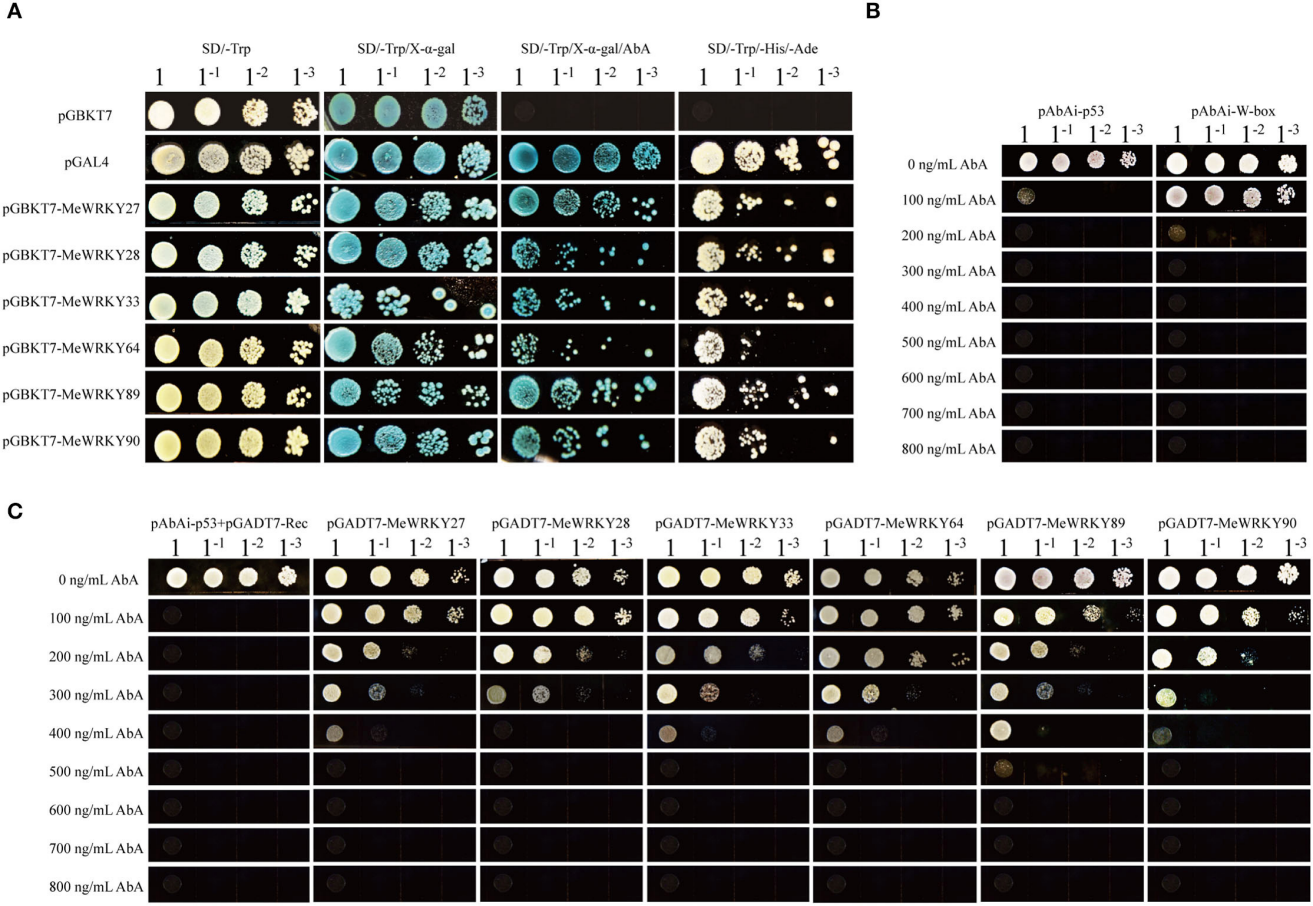
MeWRKY IIas are typical WRKY transcription factor. **(A)** Self-activation assay of MeWRKY IIas. **(B)** Determination of the minimal inhibitory concentration of AbA for pABAi-W-box. **(C)** Interaction tests between MeWRKY IIas and W-box by yeast-one-hybrid.

To understand whether the MeWRKY IIa members can serve as transcriptional activators for self-activation, we performed transcriptional activity assay. Yeast strain Y2H carrying pGBKT7-*MeWRKY IIas* vectors, respectively, were able to grow on SD/-Trp, SD/-Trp/AbA, SD/-Trp/-His/-Ade, and turned blue when X-α-gal was added, just like positive control pGAL4 (Figure 6A). These results showed that MeWRKY IIas were transcriptional factor and could auto-activate the expression of report gene.

WRKY transcript factor can bind to the W-box element on the promoter of target genes. To test the combination of MeWRKY IIas with W-box, we performed yeast-one-hybrid, where W-box was combined with pABAi vector, and pGADT7-*MeWRKY IIas* were expressed in the yeast strain Y1H-W-box (Figure 6B). The recombined yeast strains were grown on SD/-Leu medium with AbA of concentration gradients. The growth of yeast strains carrying pGADT7-*MeWRKY27/33/64/90* could be inhibited by 500 ng/ml of AbA, and the ones with pGADT7-*MeWRKY28* and pGADT7-*MeWRKY89* were inhibited by 400 and 600 ng/ml, respectively (Figure 6C). The control yeast strain with pABAi-*p53*+pGADT7-*Rec* was repressed by 200 ng/ml. These observations demonstrated that all the six *MeWRKY IIas* were able to bind to W-box, but their binding ability might vary.

### *MeWRKY IIas* Improved the Resistance to *Pst* DC3000 in *Arabidopsis*

**Figure 7:**
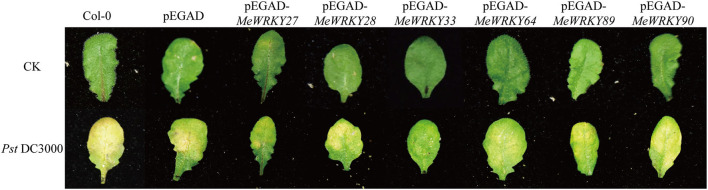
Phenotype of *MeWRKY IIa*s overexpressed *Arabidopsis* leaves inoculated with *Pst* DC3000. The transgenic *Arabidopsis* leaves with the same growth state were infected with 10 mM MgCl_2_ (CK) and *Pst* DC3000.

To explore the function of *MeWRKY IIas* in plant disease resistance, *Arabidopsis* transgenic plants overexpressing six *MeWRKY IIas* were generated. The positive transgenic plants were inoculated with *Pst* DC3000 bacteria suspension and 10 mmol/L of MgCl_2_ solution. Col-0 and pEGAD transgenic plants were used as negative controls. After 4 days, Col-0 and *Arabidopsis* plants with pEGAD empty vector showed disease symptoms with leaves turning yellow in large area (Figure 7), but leaves with *MeWRKY 27* and *MeWRKY33* overexpressed showed few yellow specks, whereas the phenotype of other *MeWRKY IIas* overexpression plants was not obvious. These indicated that the overexpression of *MeWRKY IIas* contributed to the defense against pathogen in *Arabidopsis* differently.

### Functional Deficiency of *MeWRKY IIas* Altered Disease Resistance in Cassava

**Figure 8:**
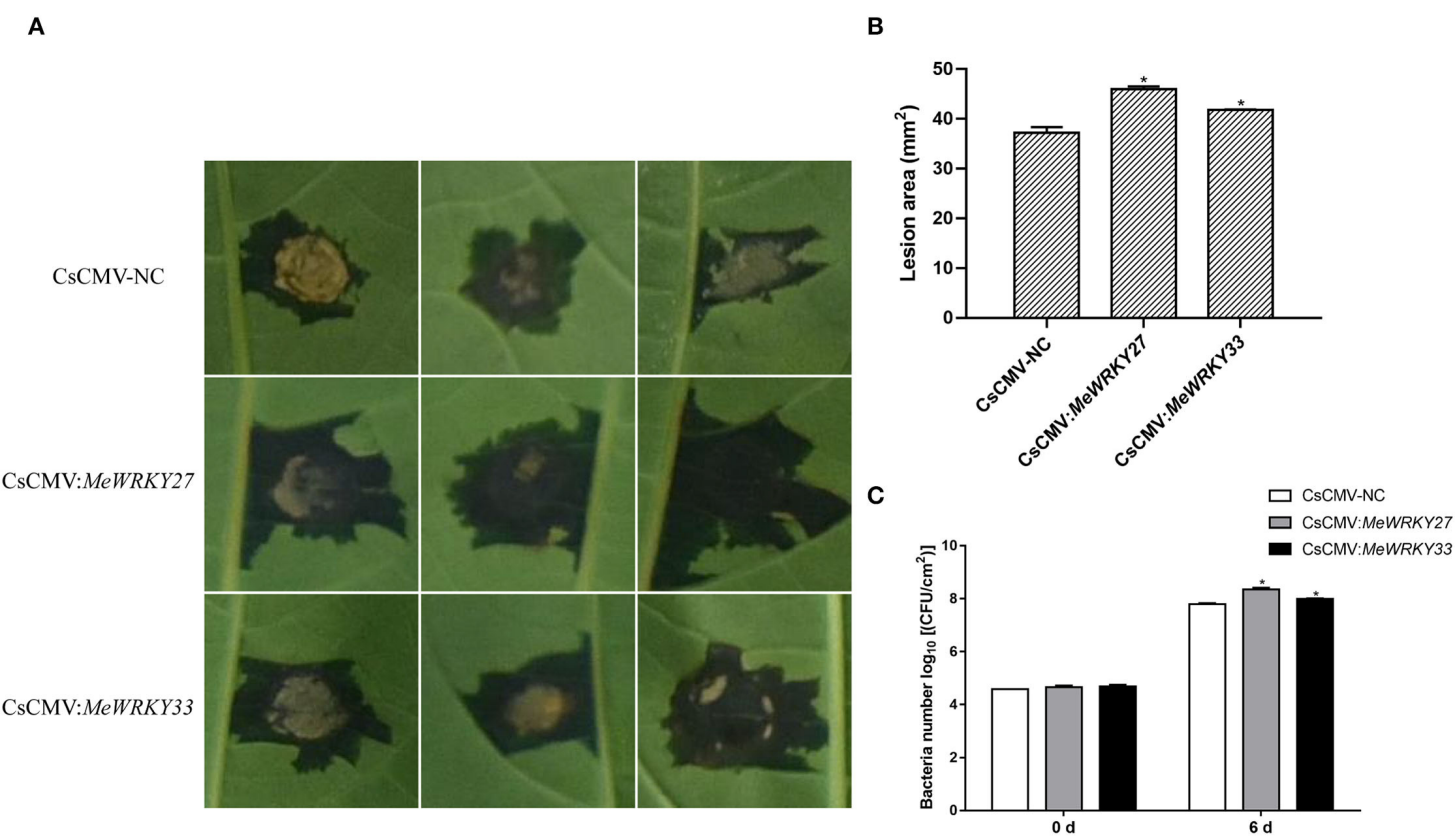
Enhanced susceptibility of *MeWRKY27* or *33*-silenced cassava leaves to *Xam* infection. **(A)** Disease symptoms on *MeWRKY27* or *33*-silenced cassava leaves at 6 days after inoculation with *Xam*CHN11 (1 × 10^8^ CFU ml^−1^). **(B)** Lesion areas caused by *Xam*CHN11. **(C)** Bacterial growth in *Xam*-infected leaves (1 × 10^7^ CFU ml^−1^). Asterisks indicate a significant difference (*p* < 0.05) between control and treatments.

As *MeWRKY27* and *MeWRKY33* positively regulate the disease resistance of *Arabidopsis*, we further investigate the function of *MeWRKY27* and *33* in cassava by generating *MeWRKY27* or *33*-silenced plants using CsCMV VIGS system. The plants infected with CsCMV:*ChlI*_345_ were used as positive control and the ones with pCsCMV-NC empty vector as negative control. After 4 weeks, the positive control plants developed severe photobleaching or a yellowing VIGS phenotype in the stems (Supplementary Figure 6A), the expression level of target genes in all plants was detected using qRT-PCR. The results showed that the expression of endogenous genes was downregulated in all virus-induced silenced plants, and the maximum was downregulated by almost 60% compared with those in the CsCMV-NC-infected leaves (Supplementary Figure 6B). Leaves of empty vector (CsCMV-NC) or silenced (CsCMV:*MeWRKY27* or CsCMV:*MeWRKY33*) cassava plants were inoculated with *Xam*CHN11. The function of *MeWRKY IIas* in defending *Xam* was determined by the area of water stain speck. We observed that silencing of *MeWRKY27* and *MeWRKY33* in cassava plants increased its susceptibility to *Xam* infection (Figures 8A,B). The lesions of silenced plants were significantly larger than the negative control. *Xam* growth in *MeWRKY27* or *33* silenced leaves was also significantly higher than in empty-vector control leaves at 6 days after inoculation (Figure 8C). Collectively, these results indicate that *MeWRKY27* and *MeWRKY33* are required for cassava defense resistance against *Xam* infection.

### Identification of Candidate Genes Regulated by *MeWRKY IIas*

**Figure 9:**
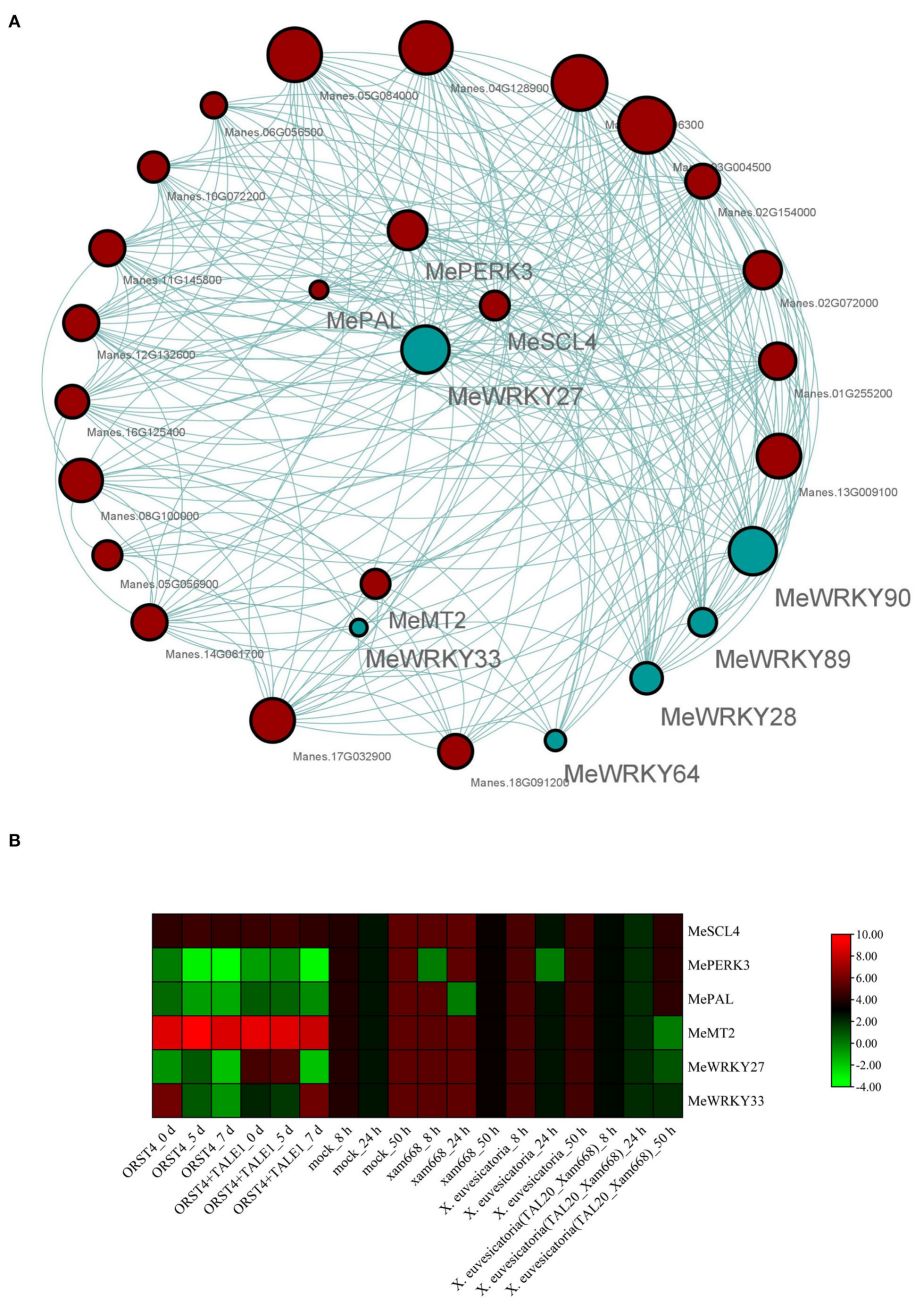
Co-expression analysis of *MeWRKY IIas* and W-box gene. **(A)** The co-expression network of *MeWRKY IIas* and W-box genes in the turquoise module. Blue and red circles represent *MeWRKY IIas* and W-box containing genes, respectively. **(B)** Expression profiles of *MeWRKY IIas* and selected W-box genes in response to various pathogen treatments.

Co-expression analysis is a powerful approach for investigating expression correlation among different genes. To further investigate the relationship between W-box genes and the selected *MeWRKY IIa* genes, about 156 genes were screened as harboring a W-box cis-element in their 2-kb promoter region (Supplementary Table 6). Co-expression analyses were performed based on 37 transcriptomes generated from *Xam*-infected cassava leaf samples. The FPKM values of the 6 *MeWRKY IIas* and 156 W-box genes in all transcriptomes were calculated and filtered. A total of 162 genes were used for co-expression analysis, whereas 13 genes were removed due to low expression or low expression variation. The gene co-expression modules for the *MeWRKY IIas* and W-box genes are shown in Figure 9A. A total of three gene co-expression modules were constructed with 41 (blue), 48 (gray), and 60 (turquoise) genes (Supplementary Table 7). All *MeWRKY IIas* were in turquoise modules. Among them, three W-box genes (*MePERK3, MePAL*, and *MeSCL4*) and *MeMT2*, which may involve in pathogenic responses, showed correlated expression patterns with *MeWRKY27* and *MeWRKY33* (weight value ≥ 0.15), respectively.

The expression pattern of selected *MeWRKY IIa* regulating genes under *Xam* infection was performed based on transcriptome data (Figure 9B). Both of them could response to *Xam* infection and showed certain similar expression pattern, especially after highly virulent *Xam*668 treatment. The functions of most of those W-box genes in cassava are largely unknown. We prepare to research the function and regulatory mechanisms of these genes to cassava disease resistance in the future experiments.

## Discussion

Members of *WRKY* family are the important transcription factors with various biological functions (Eulgem et al., 2000; Chen et al., 2018). In this study, we performed BLAST to identify the cassava WRKY family members with conserved WRKYGQK domain, which was more rigorous than the previous study with *Arabidopsis* and rice WRKY sequences as queries (Wei et al., 2016). In total, 102 *WRKY* gene sequences were identified, among which 17 new family members were not reported before. These new members enriched each known cassava *WRKY* subfamily. Compared with the number of *WRKY* genes in *Arabidopsis*, rice, and other plant species, the cassava *WRKY* genes showed no obvious expansion in the process of evolution. The lengths of ORF sequences ranging from 348 (*MeWRKY4*) to 2,226 bp (*MeWRKY65*) implied a high degree of complexity among the *MeWRKYs*. Multiple sequence alignment and phylogenetic analysis classified *MeWRKYs* into three major groups (I, II, and III) with the second group further categorized into five subgroups (IIa-IIe) based on the conserved WRKY and zinc finger-like domain. Of them, groups I and III contain 21 and 12 members, respectively, and 69 members belong to group II that is the largest group, implying that group II of *MeWRKY* genes may experience more gene duplication events during the evolutionary process. Besides, about 64% (65/102) *MeWRKYs* were found to evolve from segmental duplication events, suggesting that segmental gene duplication probably played a pivotal role in *WRKY* gene expansion in cassava genome. Gene structure and conserved motif results indicated that each MeWRKY protein was different to some extent, whereas members with same group shared a similar number of introns and similar motifs. The similar motif compositions of each MeWRKY proteins group indicated its potential functional similarity.

The heptapeptide WRKYGQK domain is highly conserved among cassava WRKY proteins, but two variations (WRKYGQR and WRKYGKK) are also identified in MeWRKY57 or MeWRKY29, MeWRKY70, and MeWRKY98, respectively. According to the previous reports (Verk et al., 2008; Zhou et al., 2008), those variations in heptapeptide WRKYGQK domain may relate to the binding specificity to W-box cis-elements, thereby suggesting that those family members have functionally diversified. Therefore, it is worthy to further investigate the functions and binding specificities of *MeWRKY29, MeWRKY58, MeWRKY70*, and *MeWRKY98*. Moreover, domain acquisition and domain loss events are a mainly divergent force for expansion of *WRKY* gene family. Among all MeWRKY proteins, MeWRKY4 had no typical WRKY domain and only had an incomplete zinc finger structure. We also found that 18 of 21 group I MeWRKYs contained two WRKY domain, whereas MeWRKY55, MeWRKY57, and MeWRKY83 had lost its N-terminal WRKYGQK-like domain. All these results implied that the *MeWRKY* genes may have experienced WRKY domain loss during the evolution.

Members from all subfamilies of WRKYs have been reported to be involved in the microbe-associated molecular pattern-triggered immunity, PAMP-triggered immunity, effector-triggered immunity, or system acquired resistance (SAR) (Chen et al., 2018). However, the study on cassava WRKY is rare. As phylogenetic analysis showed that several *MeWRKYs* clustered with *AtWRKY18, AtWRKY40*, and *AtWRKY60* in the group IIa, thereby indicating their probable common biological function, we performed a series of experiments to identify the roles of *MeWRKY IIas* played in defending bacteria. A total of seven members of the cassava *WRKY IIa* subfamily were reported here, i.e., *MeWRKY4, MeWRKY27, MeWRKY28, MeWRKY33, MeWRKY64, MeWRKY89*, and *MeWRKY90*. Compared with other subgroups, group IIa *MeWRKY* genes are much fewer. This is consistent with the fact that group IIa *WRKYs* are the subgroup with the smallest number of members in other plant species (Eulgem et al., 2000; Wu et al., 2005). All MeWRKY IIas (except for MeWRKY4) contain a single-conserved WRKYGQK domain followed by a C2H2-type zinc-finger-like motif. MeWRKY4 contained incomplete WRKY domain and was excluded in the following function research. Also, a putative leucine zipper motif, which was proposed to mediate dimerization and increase the DNA-binding affinity of WRKY proteins, was present at the N terminus of MeWRKY IIa proteins (Eulgem et al., 2000). In addition to the conserved structure of MeWRKY IIas among species, all MeWRKY IIas were observed in nucleus, had self-activation function, and were able to bind W-box in the promoter region. All these characteristics of WRKY IIas are consistent with those observed in other species (Xu et al., 2006; Jiang et al., 2012; Raineri et al., 2015). Those results suggest that the functions of *MeWRKY IIa* genes are related to the expression regulation of target genes. Thus, MeWRKY IIas are typical WRKY transcription factors and have regulatory function by binding the W-box in the promoters of other genes.

The data from transcriptome revealed that six *MeWRKY IIas* were all regulated by *Xam*, and the expression profile of each *MeWRKY IIa* under *Xam* with high virulence was different from low virulence. We also verified the result by qPCR in cassava leaves treated with *Xam*. The result indicated that except for *MeWRKY64*, all *MeWRKY IIas* showed similar expression profile that upregulated first and then declined when infected by *Xam* whereas *MeWRKY64* responded to *Xam* from 4 days. In summary, six *MeWRKY IIas* members displayed response to *Xam* infection and may the play roles in defending pathogen.

To defend various pathogens, plants have evolved complex defense mechanisms to protect themselves from pathogens and survive under changing environment, among which plant hormone transduction network is an important part (Berens et al., 2017). Hormones, JA and SA, played the vital roles in the interaction of plant and pathogens. WRKY genes were involved in the bacteria resistance in plants modulated by SA and JA. For example, pepper *CaWRKY40* are regulated by SA, JA signaling, and coordinate responses to *R. solanacearum* attacks (Dang et al., 2013). Cotton *GhWRKY15* plays a role in resistance to viral and fungal pathogens *via* SA- and JA-dependent defense pathways (Yu et al., 2012). In this study, we investigated the expression patterns of *MeWRKY IIas* under the treatment of JA and SA. *MeWRKY IIas* showed differently upregulated expression. Thus, we concluded that *MeWRKY IIas* might be involved in pathogen defense *via* several hormone pathways.

Then, we designed experiment on gain-of-function and loss-of-function plants to verify the function of *MeWRKY IIas*. First, we generated transgenic *Arabidopsis* with ectopic expression of 6 *MeWRKY IIas* and found that overexpression *MeWRKY27* and *MeWRKY33* obviously enhanced the resistance of transgenic *Arabidopsis* to *Pst* DC3000 infection. This result demonstrates that *MeWRKY27* and *MeWRKY33* may function as positive regulator in defending *Pst* DC3000 inoculation. Consistent with this, *MeWRKY27-* and *MeWRKY33*-silenced cassava plants showed reduced resistance to *Xam*.

Many WRKY transcription factors possess the dual roles in the defense. For instance, *AtWRKY53* negatively affects plant defense against *R. solanacearum* while positively regulating plant resistance to *P. syringae* (Murray et al., 2007; Hu et al., 2008). A total of three *AtWRKY IIas* members *AtWRKY18, AtWRKY40*, and *AtWRKY60* have redundant roles in response to pathogen whereas *AtWRKY18* plays a more important role than the other two (Xu et al., 2006). Besides, it has been well-explained AtWRKY18, AtWRKY40, and AtWRKY60 interacted with themselves through leucine zipper and formed homologous or heterologous dimers to change the resistance to pathogen (Xu et al., 2006). As we have proved that *MeWRKY27* and *MeWRKY33* played the positive roles in resistance to *Xam, MeWRKY IIa* genes, especially *MeWRKY27* and *MeWRKY33*, can be used as the candidate genes for cassava disease resistance breeding against CBB. On the other hand, it is worthy to further investigate the function of *MeWRKY27* and *MeWRKY33* to other cassava pathogens. Besides, we boldly infer that in cassava, *MeWRKY IIa* members may also perform redundant function in resistant to pathogen invading. It is interesting to explore that whether such physically interaction phenomenon could exist between MeWRKY27 and MeWRKY33 or with other MeWRKY IIas to form homo- or hetero-dimers.

WRKYs in other species have been proved to take part in other growth and development processes, such as seed development, dormancy and germination (Luo et al., 2005; Jiang and Yu, 2009; Ding et al., 2014), plant root development (Zhang et al., 2008), leaf senescence (Ricachenevsky et al., 2010), flowering time (Li et al., 2016; Yu et al., 2016), and so on. Based on the transcriptome data, *MeWRKY* genes showed tissue-specific expression profiles in cassava tissues at different developmental stages. Many genes from groups I, IId, and III expressed highly in cassava roots, especially the early stage of root development, indicating that they may play a crucial role in cassava root growth and development. Therefore, we speculated that *MeWRKYs* may work in cassava growth and development, and all *MeWRKYs* function together in a complicated signal network to keep growth defense in a balanced way.

WRKY transcription factors could recognize and bind to the W-box *cis*-elements of target to regulate different physiological responses. WGCNA has been used widely to identify gene modules correlated with the identification of putative transcriptional regulation in several plants (Li et al., 2019; Kesel et al., 2020; Xu et al., 2020). Our laboratory also previously reported that six *MeERFs* were significantly associated with two GCC-box genes, *MeTFIIE* and *MeASHR1* during pathogen response using WWGCNA analysis (Hong et al., 2021). Therefore, based on the principle that genes with close functional relationships or distributed in related pathways may have similar expression profiles (Lin et al., 2019), WGCNA was established to construct correlation gene network between *MeWRKY IIas* and W-box genes. Several W-box genes have been identified, including *MePERK3, MePAL, MeSCL4*, and *MeMT2*. These W-box genes may involve in disease response together with *MeWRKY IIas*. For example, phenylalanine ammonia-lyase (PAL) catalyzes the non-oxidative deamination of phenylalanine to trans-cinnamate and plays an important role in plant defense by involving in the biosynthesis of salicylic acid (SA). Pepper *CaPAL1* acted as a positive regulator of SA-dependent defense signaling to combat microbial pathogen *via* its enzymatic activity in the phenylpropanoid pathway (Sung Kim and Kook Hwang, 2014). Rice *OsPAL4* was also associated with broad spectrum disease resistance (Tonnessen et al., 2015). Their functions in cassava disease resistant will be further elucidated in the future work.

## Conclusion

In this study, a total of 102 *WRKY* genes were identified from cassava genome and further classified into three main subgroups. The expression profiles of *MeWRKYs* under pathogen infection were analyzed based on several transcriptome databases. Furthermore, the expression pattern of *MeWRKY IIas* was verified by qPCR after JA, SA, and *Xam* treatment. Gain-of-function and loss-of-function assays showed that the *MeWRKY IIas* play an important role in cassava disease resistant. Co-expression network analysis showed that different downstream genes regulated by different *MeWRKY IIa* members. Collectively, our results provide a theoretical basis for further understanding of the molecular response of *MeWRKY IIas* to pathogen infection in cassava. In addition, it is also helpful for future genetic improvement and breeding.

## Data Availability Statement

The original contributions presented in the study are included in the article/Supplementary Material, further inquiries can be directed to the corresponding authors.

## Author Contributions

SZ: writing-original draft, investigation, and data curation. RF: software, methodology, and visualization. XX: investigation and data curation. JL, LX, YH, and YY: visualization and validation. XZ and XY: conceptualization, writing, reviewing, and editing. YC: conceptualization, writing, reviewing, editing, supervision, and funding acquisition. All authors have read and approved the final manuscript.

## Funding

This work was supported by the grants from the National Key Research and Development Program of China (2018YFD1000500), Key R&D Program of Hainan province (ZDYF2019063), China Agriculture Research System (CARS-11-hncyh).

## Conflict of Interest

The authors declare that the research was conducted in the absence of any commercial or financial relationships that could be construed as a potential conflict of interest.

## Publisher's Note

All claims expressed in this article are solely those of the authors and do not necessarily represent those of their affiliated organizations, or those of the publisher, the editors and the reviewers. Any product that may be evaluated in this article, or claim that may be made by its manufacturer, is not guaranteed or endorsed by the publisher.

## Supplementary Material

Supplementary Figure 1 Distribution of *MeWRKY* genes on cassava chromosomes. Vertical bars represent chromosomes. Base-pair positions are indicated on the left.

Supplementary Figure 2 Unrooted phylogenetic tree of WRKY proteins from cassava and Arabidopsis. The NJ tree was constructed using MEGA 7.0 with 1,000 bootstraps.

Supplementary Figure 3 Multiple sequence alignment of the WRKY domain among cassava MeWRKYs and selected AtWRKYs. A total of seven different Arabidopsis WRKY proteins from each of the groups or subgroups were randomly selected as representatives for the further comparison. For group I WRKY proteins, “N” and “C” indicated the N-terminal and C-terminal WRKY domains of a specific WRKY protein, respectively. The typical amino acid residues within WRKY domain and zinc-finger motif were in red and blue color, respectively. The conserved introns were indicated by arrowhead.

Supplementary Figure 4 Multiple sequence alignment of cassava WRKY IIa members.

Supplementary Figure 5 Expression profiles of cassava *WRKYs* in leaves and roots of Argentina 7 (Arg7), wild subspecies (W14), and Kasetsart University 50 (KU50).

Supplementary Figure 6 VIGS phenotype of cassava leaves. **(A)** Silencing phenotypes on cassava leaves at 30 dpi using CsCMV VIGS system. pCsCMV-*ChlI*_345_ was used as positive control. **(B)** RT-qPCR analyses of target gene expression levels in VIGS plants.

Supplementary Table 1 Primers used in this study.

Supplementary Table 2 Transcriptome datasets used for expression and WGCNA analysis.

Supplementary Table 3 The detailed information of *MeWRKY* genes identified in the cassava genome.

Supplementary Table 4 Gene duplication events of *MeWRKYs*.

Supplementary Table 5 Sequence logos of the conserved motifs in cassava WRKY proteins.

Supplementary Table 6 Summary of the annotation for 156 cassava genes containing W-box in their 2-kb promoter region.

Supplementary Table 7 Genes belong to different co-expression modules built from co-expression analysis.
