# Supplementary figures and images for: MeWRKY IIas, Subfamily Genes of WRKY Transcription Factors From Cassava, Play an Important Role in Disease Resistance
Source: Front Plant Sci. 2022 Jun 2;13:890555. doi: 10.3389/fpls.2022.890555 (PMC9201764; doi:10.3389/fpls.2022.890555)

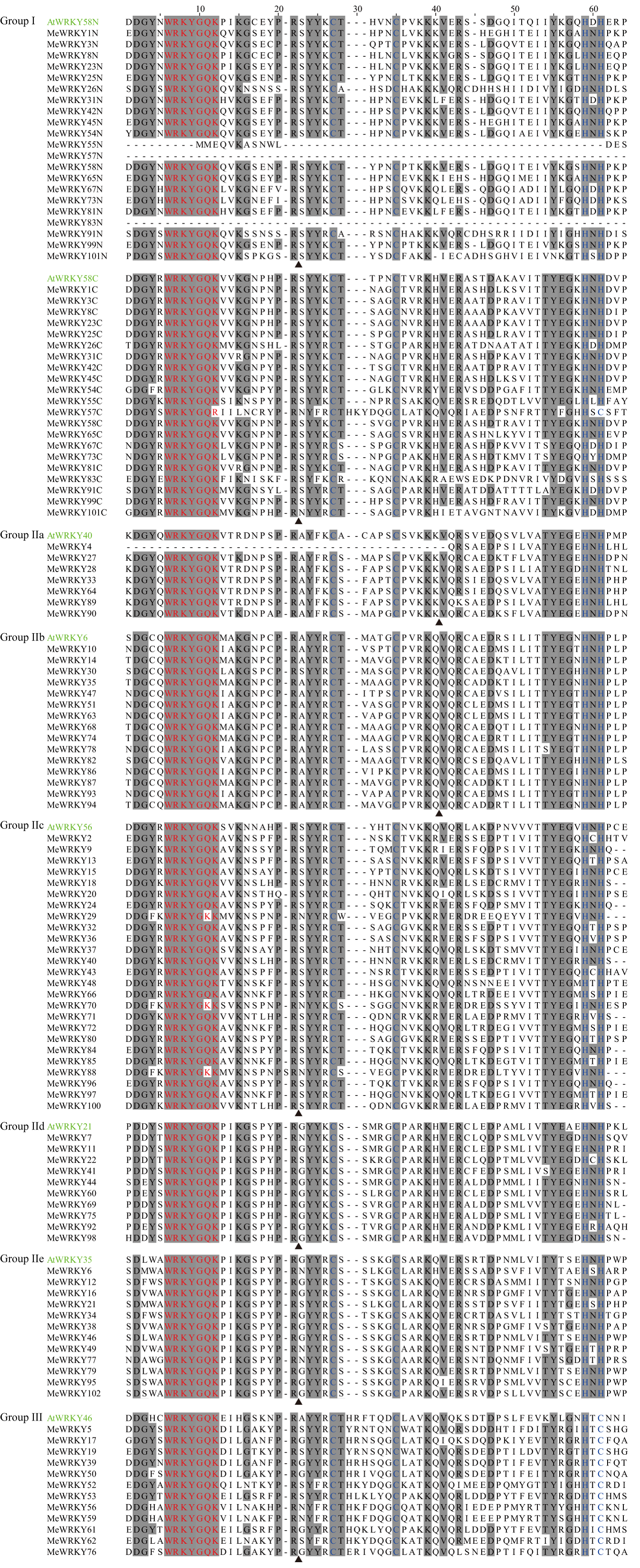

Supplement: Supplementary Figure 3 — Multiple sequence alignment of the WRKY domain among cassava MeWRKYs and selected AtWRKYs. A total of seven different Arabidopsis WRKY proteins from each of the groups or subgroups were randomly selected as representatives for the further comparison. For group I WRKY proteins, “N” and “C” indicated the N-terminal and C-terminal WRKY domains of a specific WRKY protein, respectively. The typical amino acid residues within WRKY domain and zinc-finger motif were in red and blue color, respectively. The conserved introns were indicated by arrowhead. [file Image_3.TIF]

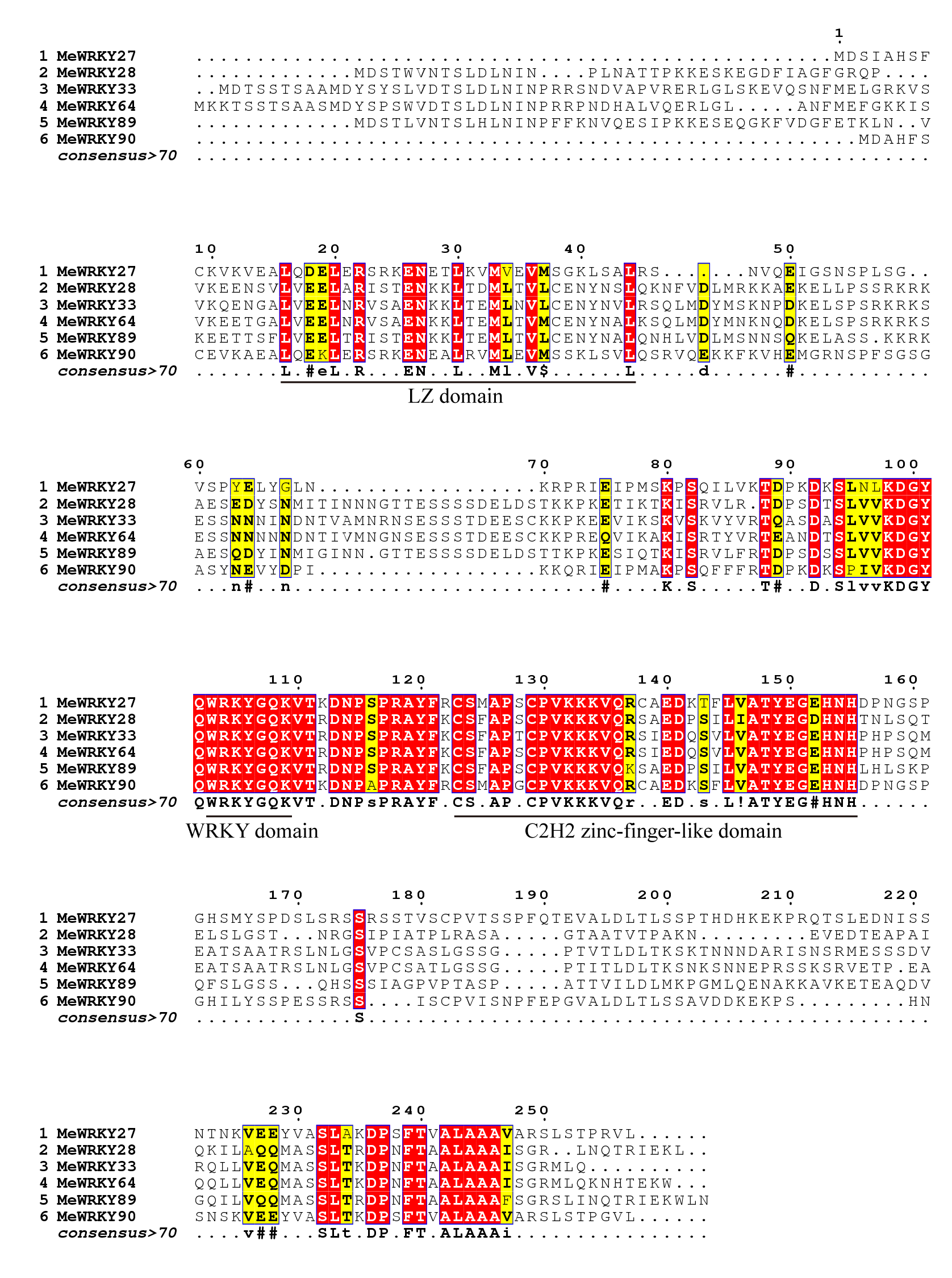

Supplement: Supplementary Figure 4 — Multiple sequence alignment of cassava WRKY IIa members. [file Image_4.TIF]

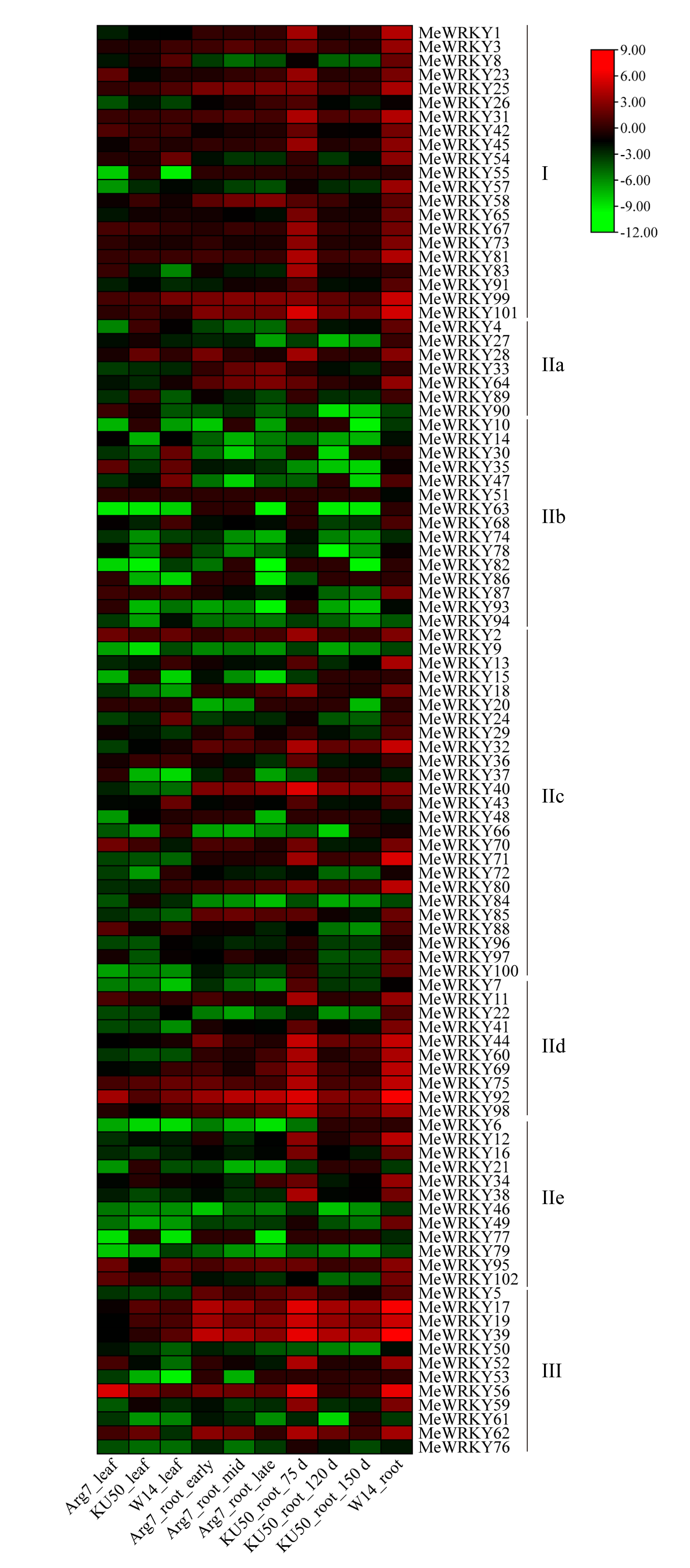

Supplement: Supplementary Figure 5 — Expression profiles of cassava WRKYs in leaves and roots of Argentina 7 (Arg7), wild subspecies (W14), and Kasetsart University 50 (KU50). [file Image_5.TIF]

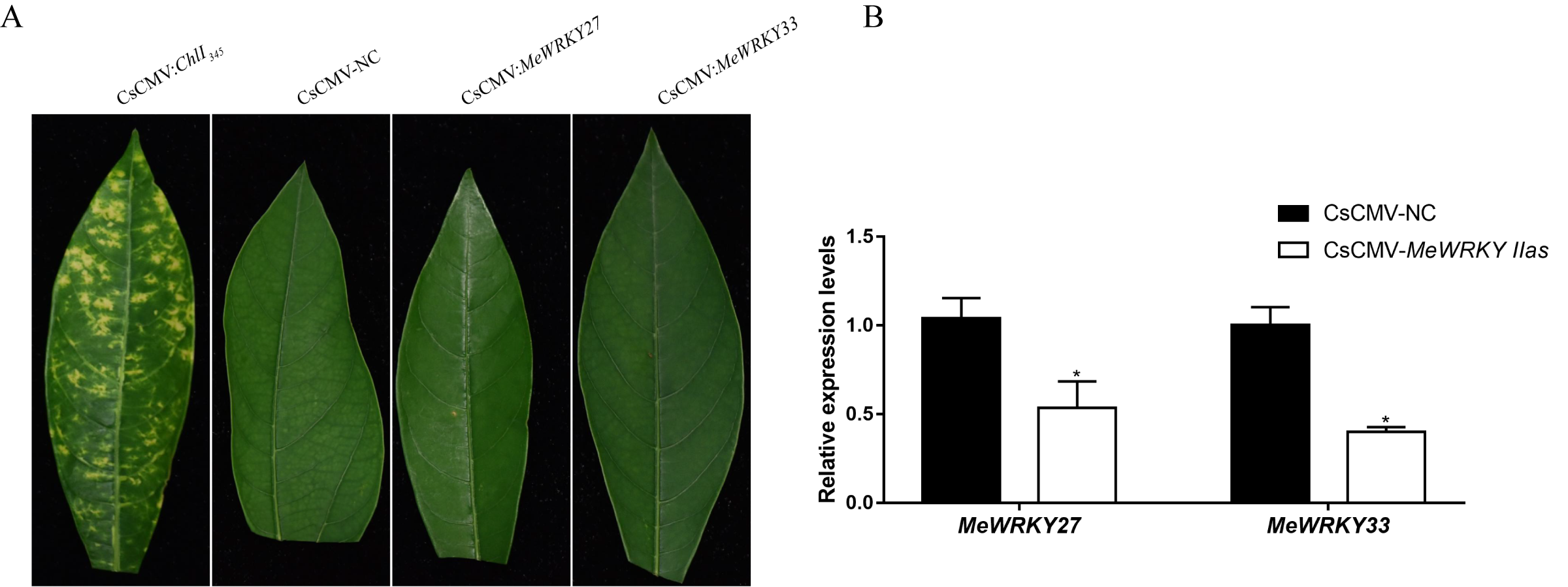

Supplement: Supplementary Figure 6 — VIGS phenotype of cassava leaves. (A) Silencing phenotypes on cassava leaves at 30 dpi using CsCMV VIGS system. pCsCMV-ChlI345 was used as positive control. (B) RT-qPCR analyses of target gene expression levels in VIGS plants. [file Image_6.TIF]
